# The native aortic valve reduces paravalvular leak in TAVR patients

**DOI:** 10.3389/fphys.2022.910016

**Published:** 2022-08-05

**Authors:** Anthony R. Prisco, Jorge Zhingre-Sanchez, Lars Mattison, Demetris Yannopoulos, Ganesh Raveendran, Paul A. Iaizzo, Sergey Gurevich

**Affiliations:** ^1^ Department of Medicine, Division of Cardiology, University of Minnesota, Minneapolis, MN, United States; ^2^ Department of Surgery, Visible Heart Laboratories, University of Minnesota, Minneapolis, MN, United States; ^3^ Department of Biomedical Engineering, University of MN, Minneapolis, MN, United States

**Keywords:** transcatheter valve replacement, 3D printing, paravalvular leak, computational fluid dynamics, paravalvular leak

## Abstract

**Background:** Paravalvular leak (PVL) is a frequent TAVR complication. Prospective identification of patients who are likely to develop PVL after TAVR would likely lead to improved outcomes. Prior studies have used geometric characteristics to predict the likelihood of PVL development, but prediction and quantification has not been done. One of the reasons is that it is difficult to predict the mechanical deformation of the native diseased aortic valve prior to implantation of the prosthetic valve, as existing calcifications likely contribute to the seal between the prosthetic valve and the aortic annulus. However, the relatively amount the native valve plays in preventing PVL is unknown.

**Methods:** A retrospective chart review was conducted identifying patients with mild or greater PVL. One patient who had substantial PVL was identified and a 3D printed (pre-TAVR) aortic root was created. Balloon-expandable TAVR stent frames were implanted within the 3D printed root and a new model was created. Using this geometry, computational fluid dynamics (CFD) simulations were done to quantify PVL. The PVL flow path was iteratively decreased to simulate the space occupied by a crushed native aortic valve and PVL was quantified.

**Results:** PVL was found to decrease as the space occupying the PVL area increased, demonstrating that the native aortic valve contributes to reducing regurgitation. CFD simulations demonstrated that within the patient analyzed, the native valve occupies between 3–40% of the PVL pathway.

**Conclusion:** *A priori* techniques that predict the development of post TAVR PVL should account for the native diseased valve as our simulations demonstrate that it plays a role in reducing PVL.

## Background

Transcatheter aortic valve replacement (TAVR) is poised to replace surgical aortic valve replacement (SAVR) as the standard of care across all risk groups. TAVR has been shown to be either equivocal or superior to SAVR in the majority of patients across all risk strata (Leon et al., 2010; Smith et al., 2011; Leon et al., 2016; Mack et al., 2019). Additionally, TAVR is the only available option in non-operable patients (Leon et al., 2010). Long-term durability data for TAVR are promising and this trend is projected to increase.

Despite its widespread adoption and improving outcomes, there are a number of TAVR specific complications that continue to impact long term valve durability as well as patient mortality and morbidity (Bagur et al., 2012; Van Der Boon et al., 2012). Paravalvular leak (PVL) remains a frequent complication associated with both balloon-expandable and self-expanding aortic valves (Bagur et al., 2012; Van Der Boon et al., 2012). Early generations of balloon-expandable valves such as the Sapien and Sapien XT were associated with significant PVL due to an in-stent valve design and incomplete seal of the bare metal stent frame against the calcified native valve leaflets and left ventricular outflow tract (LVOT). Short and long-term studies showed that moderate PVL placed patients at increased risk of both stroke and mortality (Kodali et al., 2012; Généreux et al., 2013; Maisano et al., 2015; Feldman et al., 2018). Second generation balloon and self-expanding valves were designed incorporating sealing skirts along the valve base that reduced the frequency and magnitude of the PVL. This proved successful in reducing PVL but almost doubled the rates of complete heart block requiring pacemaker placement (Bagur et al., 2012; Van Der Boon et al., 2012). It has been reported that pacemaker placement itself is associated with increased morbidity (Bagur et al., 2012; Van Der Boon et al., 2012).

While moderate PVL has been dramatically reduced if not completely eliminated in the majority of degenerative tricuspid cases, mild PVL remains common (Kodali et al., 2012; Généreux et al., 2013). Data from the REPRISE III trial shows that even mild PVL results in excess morbidity with a significantly higher rate of stroke than patients with no PVL (Feldman et al., 2018). Despite quantitative criteria from the Valve Academic Research Consortium guideline, PVL suffers from underestimation as there is often discordance between the measurement of PVL on fluoroscopy, echocardiography, and hemodynamic assessment *via* left ventricular end-diastolic pressure changes (Tanaka et al., 2018).

Pre-procedure computational modeling using 3D printed phantom and *in-vitro* TAVR valve implants have shown that presence/absence of PVL can be accurately predicted; more so than what would be expected based on traditional risk assessments such as annulus shape, size, and the degree of calcium burden (Reiff et al., 2020). The majority of these studies have either experimentally or virtually implanted a given valve into a 3D model. Likelihood and location of PVL is then estimated from these models. Quantification of the regurgitant volume, however, requires further mathematical modeling using simulations called computational fluid dynamics (CFD).

There are already preliminary data in the literature to suggest that mathematically derived computer simulations of valve implants are capable of accurately reproducing *in-vivo* procedures. Contemporary literature shows that mathematical modeling of Sapien XT second-generation balloon-expandable implants were successfully modeled with adequate results (Morganti et al., 2014; Bosmans et al., 2016; Schultz et al., 2016; Bosi et al., 2018). PVL locations were identified in 79% of cases and resultant diameters were similar within an error margin of 2.5 ± 3.9% (Bosi et al., 2018). While these results are an improvement, they still lack the real-world reliability that has been more successfully demonstrated with 3D printed models (Reiff et al., 2020). First, the error margin remains wider than clinically acceptable. Second, 79% detection rate of PVL remains a low bar that is easily matched by more conventional approaches such as calcium scoring and annulus size or elliptic indices. Third, the majority of these models described to date lack CFD assessment which is computationally intense but highly accurate at predicting the fluid mechanics of complex blood flows including turbulence. Interestingly, de Jaegere et al. (2016) showed in 60 patients a high correlation between CFD simulation and invasive measurements on fluoroscopy. This was limited by the use of only self-expanding prostheses and lack of methods data on patient’s anatomy and modeling. More recently, Bianchi et al. (2019) have recently rigorously described their approach to reconstruction and modeling. Using a rigorous simulation with mathematical modeling and valve deployment, they were able to show concordance with echocardiographic and fluoroscopic findings in terms of PVL location and severity. They also demonstrated changes in PVL based on valve implant depth and final valve expansion (Bianchi et al., 2019).

We have previously demonstrated that our 3D printed phantoms were capable of accurately predicting the location of subsequently observed PVL. We published our methods showing that 3D printed phantoms implanted with stent frame balloon-expandable valves can accurately predict areas of PVL beyond that of traditional risk factors such as annulus size, annulus ellipticity, and calcium volume and implant location (Reiff et al., 2020). However, this method was unable to quantitate the relative degree of regurgitant volumes. Finally, an additional area that has not been considered when using CFD to predict PVL is the contribution of the native valve to post-implantation geometry. This is difficult as the solid mechanics of the calcified aortic valve are not well understood. Here, we demonstrate that mathematical modeling using CFD can predict the location and accurately quantify the PVL. Additionally, using an iterative approach we demonstrated the relative contribution of the native valve in preventing PVL.

## Methods

### Patient selection/review

**FIGURE 1:**
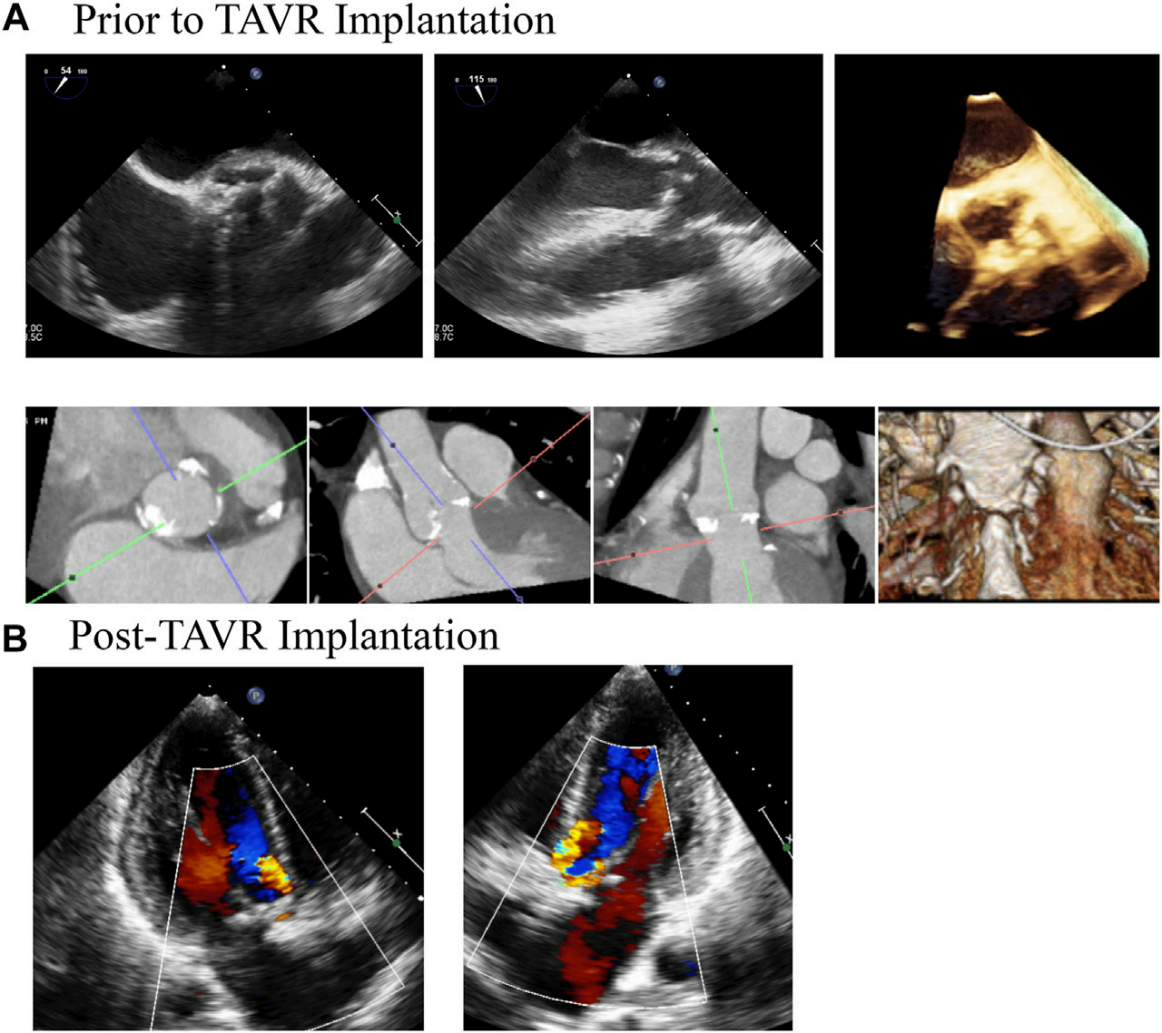
Medical Imaging of Patients Aortic Valve Demonstrates Clinically Significant Paravalvular Post TAVR Implantation. A patient was referred for TAVR for severe aortic stenosis. **(A)** demonstrates TEE and CT imaging of the aortic prior to the procedure. The patient had trace aortic insufficiency prior to the procedure. Following the procedure **(B)**, the patient was found to have moderate paravalvular regurgitation that persisted for at least 1 year following the procedure, as demonstrated by the follow up TTE.

A retrospective review of all patients who underwent TAVR implantation (between 2012–2017) was approved by the institutional review board at the University of Minnesota (No. 00004338). Patients who underwent TAVR implantation for severe aortic stenosis who also had clinically significant paravalvular leak were identified (those with at least mild PVL). Patients who had a transcatheter valve placed in a non-aortic position (mitral or tricuspid) were excluded. Additional exclusions were for valve-in-valve implants and those implanted in bicuspid valves. After de-identification, pre and post-operative imaging and blood pressures were obtained (echocardiograms, computed tomography scans), as well as the associated imaging reports. One patient was identified who met all of these criteria; pre and post-operative imaging is outlined in Figure 1.

### Geometry creation

**FIGURE 2:**
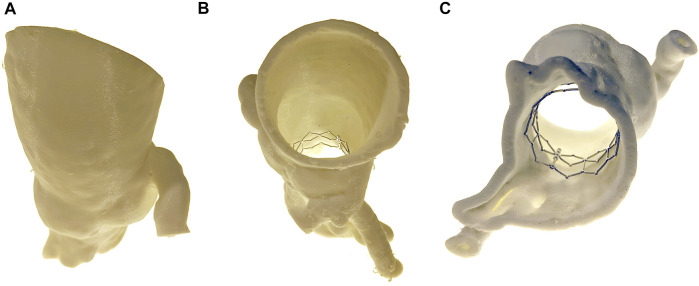
3D Printed Phantom. A 3D printed phantom was generated from the patient preoperative imaging. The anatomy was segmented from the distal end of the LVOT to the proximal aorta. A TAVR cage was then inserted into the phantom and the entire phantom was reimaged. Views include **(A)** anterior, **(B)** superior, and **(C)** inferior.

Pre-implantation gated-CT scans images (DICOM) were imported into Mimics v20.0 (Materialise Inc., Leuven, Belgium). Representative models including the blood flow path were created *via* manual segmentation from the left ventricular outflow tract, through the first 10 cm of the proximal aorta. The aortic valve leaflets were excluded. Models were converted to stereolithography files (STL) and were then printed on an Ultimaker three Extended 3D printer (Figure 2) using a material called Ninjaflex (Ninjatek, Manheim, PA). This material was selected as it is flexible and has a similar stiffness to human tissues and is consistent with previous studies (Reiff et al., 2020). A 26 mm Edwards-Sapien XT valve cage was then implanted *ex vivo* into the expected aortic position and the entire model was re-imaged in a Siemens SOMATOM flash dual source CT. Again, the DICOM files were extracted and the blood flow path was then manually segmented in Mimics v20.0. The geometry was smoothed and imported into Autodesk Fusion 360 (San Rafael, California) and inlet/outlet boundaries were created. The entire geometry was imported into Autodesk CFD 2019 (San Rafael, California) for modeling.

### Computational fluid dynamics simulations

**FIGURE 3:**
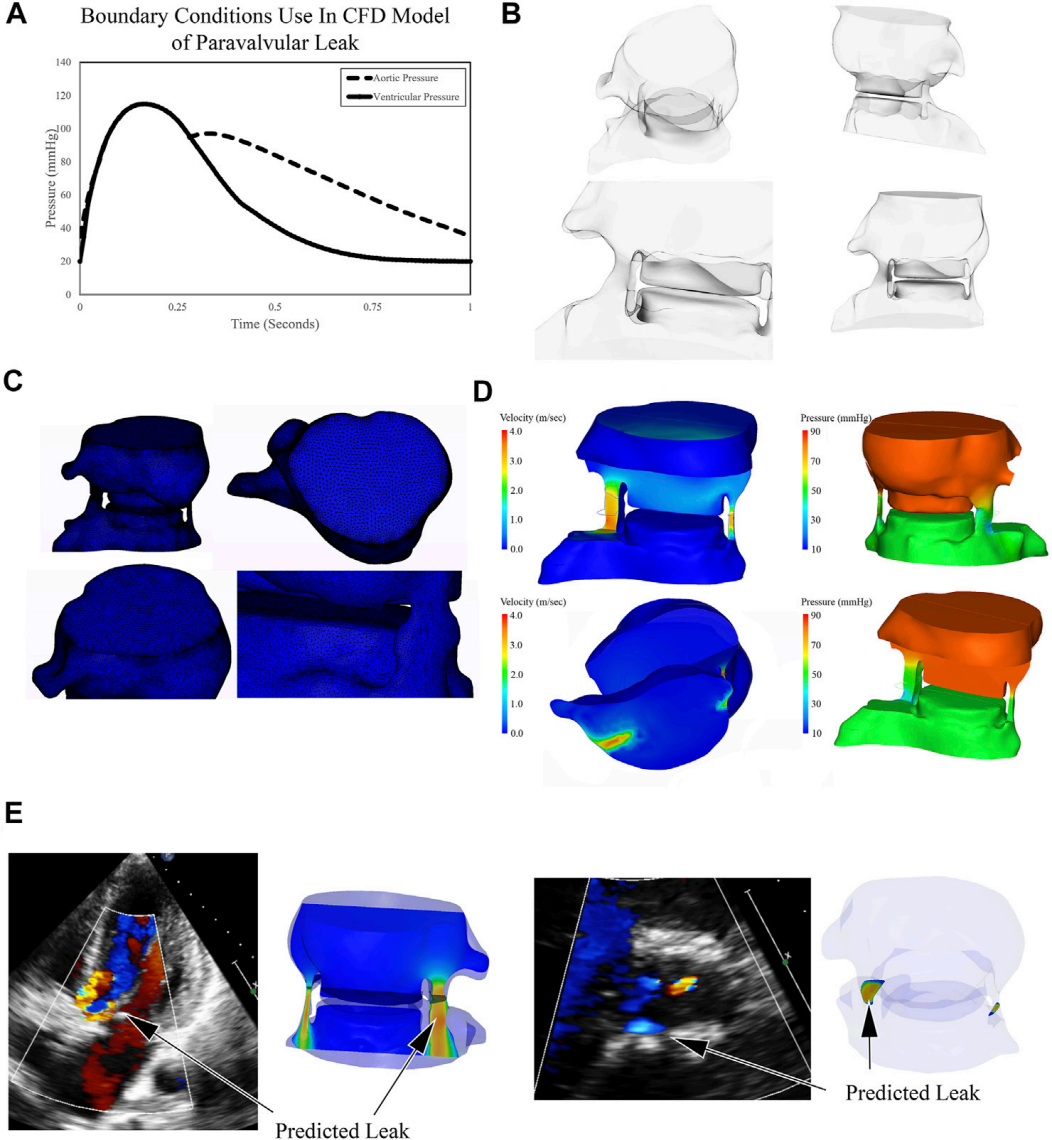
Outline of Computational Fluid Dynamics Strategy to Simulate and Quantify Paravalvular Leak. **(A)** Representative aortic blood pressure waveform was fit to the patient’s post procedural blood pressure; ventricular waveform was estimated. These waveforms were used as boundary conditions for simulations. **(B)** Post procedural blood volume was generated in CAD software and converted to a 2.5 million element mesh **(C)**. CFD solver determined the pressure and velocity values for each element and blood flow during diastole was simulated, representative images are shown **(D)**. Paravalvular leak was predicted in two areas, one large and one small. The large area of PVL was seen on follow up echo, as the smaller was likely obscured by the region of valvular insufficiency **(E)**.

After being imported into Autodesk CFD 2019, the imported geometry was converted into a rigid mesh of 2.5 million elements. Models were set up as follows:

1) Transient CFD simulations were run using the modified Petrov-Galerkin solver. Because paravalvular regurgitation only occurs during diastole, leak of a single beat was simulated during diastole for 0.7 s at a time step of 0.0005 s.
2) Blood was modeled as an incompressible Newtonian fluid with a viscosity of 0.0035 Pa-s and a density of 1060 kg/m^3^, consistent with previous studies. Fluid was modeled as turbulent, using a K-epsilon model.
3) A no-slip boundary condition was approximated for all walls within the simulation, and the LVOT and Aortic boundaries were modeled as transient pressures according to the curves in Figure 3A. Curves were representative waveforms from the aorta and left ventricle that were fit to patient data. The aortic pressure waveform was parameterized to the patient’s post-implantation blood pressure (115/36 mmHg). The LV pressure waveform was parameterized to the patient’s post-implant blood systolic pressure and estimated left ventricular end-diastolic pressure (20 mmHg).
4) In the initial geometry, the native valve leaflets were not included to allow for testing of the independent variable, percent occlusion of the paravalvular leak. Serial CFD simulations were run and the percent occlusion of the paravalvular leak was iteratively increased. Regurgitant volume was calculated by quantifying the flux through a plane drawn halfway through the pathway of the simulated paravalvular leak.

## Results

### Computational fluid dynamics simulations

The blood flow path around the aortic valve was created from the LVOT to the proximal aortic annulus (Figures 2, 3). Pressure boundary conditions were imposed on the ventricular and aortic sides of the fluid domain. The pressure boundary conditions were derived from the patient’s blood pressure measured after the TAVR procedure had been completed. On the aortic side, a representative blood pressure waveform was fit to the patient’s systolic and diastolic blood pressures (Figure 3A). On the ventricular side, the pressure waveform was fit to the patient’s systolic blood pressure and a left ventricular end-diastolic blood pressure of 20 mmHg. As the patient’s left ventricular end-diastolic blood pressure was not available, due to the high amount of regurgitation observed clinically, this value was estimated.

Because paravalvular leak is only observed during diastole, simulations were conducted from the instant the aortic valve closed throughout the rest of diastole. Simulations were then run and the regurgitant flows were calculated through each of the two regurgitant pathways. The larger of the pathway had a regurgitant flow volume of 55 ml, whereas the smaller one was 5.6 ml. It should be noted that while our technique identified two areas of PVL, there was only a single PVL jet seen on the post-operative echocardiogram. This is likely because the patient had a mild degree of valvular insufficiency that prevented detection of the smaller of the two PVL regions. When the two regions are added together, the regurgitant volume borders on severe PVL (Regurgitant Volume >60 ml), however, the patient’s post-operative echocardiograms were consistently in the “moderate” range. The discrepancy is likely driven by the absence of the native leaflets in our simulations. The presence of the leaflets would increase the resistance to flow, reducing the gradient between the two boundaries. This results in decreased flow across the lesion and reduces the predicted regurgitation.

The 3D printed phantom created for this study did not include the native aortic leaflets. While it is known that the native leaflets are crushed as the TAVR prosthesis is deployed, the mechanical interactions between the aortic valve and the stent frame apparatus is not well understood. Thus, as the initial simulations likely overestimated the regurgitant fraction, the remaining simulations were designed to test the hypothesis that the presence of the native valve would decrease the volume of regurgitant flow.

### Filling paravalvular leak void

**FIGURE 4:**
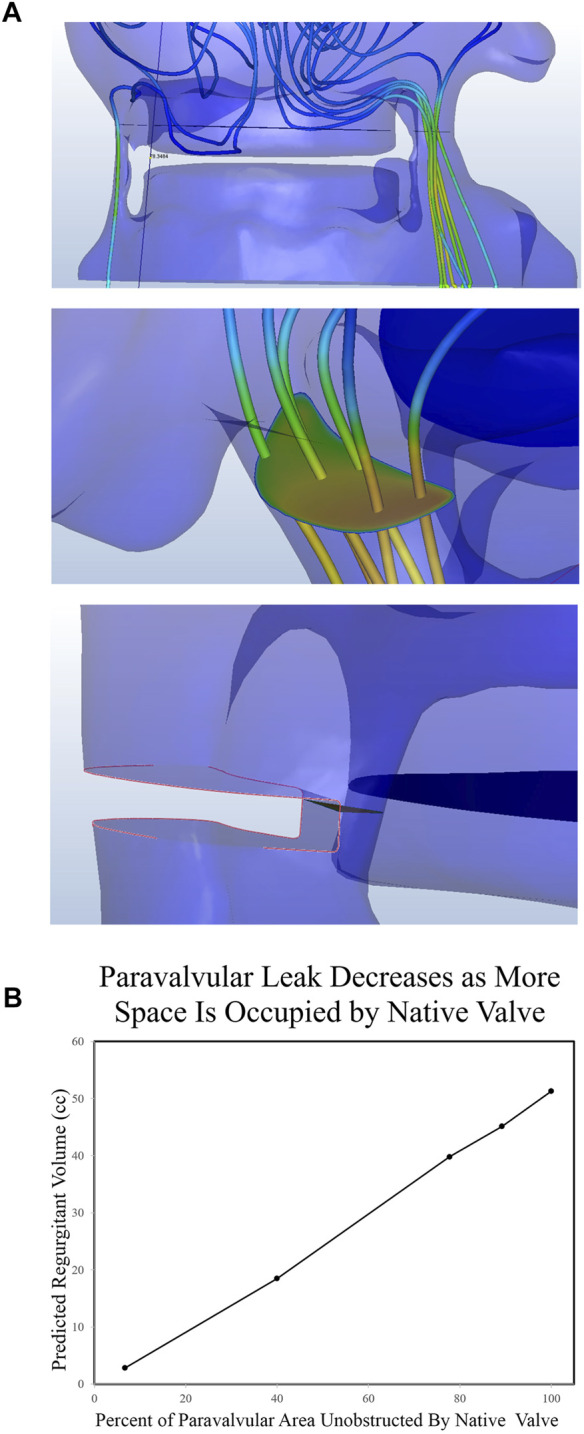
Quantification of Paravalvular Leak from CFD Simulations. **(A)** Post implantation imaging demonstrated two clinically significant leaks, both of which were recreated in the CAD software. A plane was drawn midway through the leak area and the flux through that plane was quantified. Finally, portions of the paravalvular area were sequentially occluded and leak was quantified again. **(B)** The regurgitant volume was then quantified as a function of the percent paravalvular area that was occluded.

To test the hypothesis that regurgitant flow would be impeded by the presence of the native aortic valve, we designed a series of simulations where the cross-sectional area of the regurgitant pathway was iteratively reduced for each successive simulation. The diastolic regurgitant volume was quantified as a function of the percent simulated occlusion to the regurgitant flow path. This was done by inserting a solid area into the regurgitant geometry (Figure 4A) at the narrowest portion of the flow path. As this was done a first-order linear relationship was observed between the percentage of occluded flow path and the volume of diastolic regurgitation (Figure 4B). While it is intuitively obvious that the native valve will take up some portion of the regurgitant area, the relative percentage is unclear. Post procedure, 30 days, 6 months, and 12 months echocardiograms were obtained. The degree of PVL was rated as moderate in severity on all of the post procedure imaging. Regurgitant volume was not quantified, but moderate aortic regurgitation is generally accepted as 30–59 ml (Zoghbi et al., 2019). Our total calculated regurgitant volume of 60.6 ml (prior to adding native aortic valve leaflets) demonstrates that the native aortic valve occupies a portion of the flow path. Because the patient’s PVL was rated in the “moderate” range, the native valve does not occupy more than 40% of the cross-sectional area in the regurgitant flow path (Figure 4B).

## Discussion

In this study, we identified a patient that underwent a balloon expandable TAVR which post-procedurally was identified with moderate PVL. Briefly, using previously published 3D printing methods, we obtained a preoperative CT scan to generate a 3D printed phantom which was then implanted with the same size stent frame for the patient in question. Fluoroscopic and echocardiogram comparison was made to ensure identical positioning of the valve frame as in the clinical case with respect to depth and balloon inflation. The 3D printed phantom was then converted to a digital phantom as previously published (Reiff et al., 2020). The digital phantom was then analyzed visually for location of the PVL which was confirmed with echocardiographic and fluoroscopic data. Mathematical modeling was then performed using CFD simulations to quantify the degree of PVL. Our simulations showed accurate prediction of the location of the PVL as detailed previously. CFD simulations demonstrated a regurgitant volume similar to the patient’s post-operative echocardiograms when the valve PVL cross sectional area was 3–40% occluded (this includes the range of moderate PVL encompassing any regurgitant volume >30 ml up to 60.7 ml), suggesting that after the native valve was crushed, this was equivalent to the cross sectional area it occupied in that region. This is the first study that investigated the relative contribution of the native leaflets in preventing PVL.

Studies that have attempted to predict the likelihood of developing PVL have used a combination of simple geometric analysis, 3D printing, and mathematical modeling to determine if a given patient will develop clinically significant PVL. The majority of these studies are non-quantitative, rather, they simply predict absence/presence of PVL. To make a quantitative prediction of the degree of PVL, it is necessary to simulate the fluid mechanics of blood flow in diastole using computational fluid dynamics. Small inaccuracies in the geometry used for simulation can significantly alter the blood flow behavior resulting in an incorrect solution. While modern CT scanners provide enough anatomical detail to predict blood flow through the aorta, during the TAVR procedure, the native aortic valve is crushed and pushed to the side of the TAVR prosthesis. The mechanics of this behavior is not understood. We tested the hypothesis that the crushed native valve geometry plays a role in preventing PVL. This was done by simulating PVL in the absence of a native aortic valve, and then iteratively adding material to the PVL pathway as a model of the crushed native valve. Simulations suggest that the native valve plays a role in preventing PVL and future methods should take this into consideration when predicting PVL.

Previous studies from our group have shown that our digital phantoms mimic *in-vivo* implantations of TAVR valves including mechanical complications associated with the implant (Reiff et al., 2020). While 3D printed phantoms provide valuable insight towards the anatomical constraints of a patient, from the geometry alone it is difficult to predict an estimated magnitude of the resultant PVL. CFD modeling bridges the gap between anatomic and functional analysis and accurately quantifies the severity of PVL after accounting for the crushed native valve leaflets. After providing a reasonable estimate for the contribution of the native valve leaflets to the PVL area, our CFD model was able to accurately demonstrate the location of the PVL when compared to post implant echocardiographic findings and 3D printed phantoms. This has previously been shown to be possible using pure *in silico* studies (Bianchi et al., 2019). However, those models rely on multiple mathematical assumptions and have only been validated in small cohorts. In larger cohorts, digital implantation of valves using mathematical models falls short of *in vivo* clinical expectation (Bosi et al., 2018). Our models have shown high sensitivity and specificity in detecting PVL with *ex-vivo* implants in 3D printed phantoms (Reiff et al., 2020).

As TAVR has been FDA approved as a therapeutic option in all aortic stenosis risk groups, there are still complications that are unique to patients who received treatment *via* TAVR. One of those is PVL. As next generation valve designs have reduced the incidence of PVL, even mild PVL is associated with increased morbidity and stroke. Prediction of patients who will have significant PVL will likely reduce PVL associated morbidity. Prediction requires in depth analysis of the patient’s aortic anatomy prior to vale implantation. A number of other groups have investigated PVL and have attempted to make *a priori* predictions using techniques similar to ours. One aspect that has largely been ignored is the contribution of the native valve to creating a seal between the prosthetic and patient’s aortic annulus. This is important, as the volume occupied by the calcified native valve is non-trivial. In the patient modeled in this paper, there were two areas of PVL, a large one and a small one.

When the initial CFD analysis was conducted, the native valve was completely omitted, demonstrating a regurgitant volume of >60 ml (severe PVL). This demonstrates that the native valve plays a significant role in preventing PVL, especially in situations where annular ellipticity index is high (Reiff et al., 2020). Because the solid mechanics of native valve balloon deformation are not yet predictable, we included material that impeded the flow path in our models (Figure 4). When occupying at least 3% of the PVL flow path, the severity of the PVL was consistent with post-operative echocardiographic findings. Since the range of regurgitant volume of moderate PVL extends from 30 to 59 ml, we can estimate that the native leaflets contribute at least three and up to 40% of the occupying space. These results demonstrate that the native valve plays a significant role in preventing PVL. Further work can be done to predict the deformation characteristic and how the native valve will affect flow after balloon dilation.

Finally, this work demonstrates the importance of native leaflets in PVL. This is important as it shows that procedures aimed at deforming or lancing leaflets such as the BASILICA (bioprosthetic or native aortic scallop intentional laceration to prevent iatrogenic coronary artery obstruction) procedure may inadvertently increase risk for PVL. Khan et al. (2019) showed that in post BASILICA patients, thermal damage from electrocautery with destruction of the entire native leaflet is possible. Given the important impact of native leaflets on PVL, this can be another significant predictor in post TAVR PVL. While we have shown this impact in only one patient, our study lays the groundwork for further investigation as these procedures become more common.

There are a number of limitations in our study. First, this is a single patient proof of concept study and these results need to be validated in a larger cohort of patients. Second, the CFD design has been modeled using a balloon-expandable valve and therefore cannot be extrapolated to self-expanding valves without further study. Third, native valve leaflets were removed from the aortic roots prior to construction of the 3D phantoms and the digital phantom models. The leaflets were approximated as simple blocks of tissue in post stent frame implants, simplifying *in vivo* geometry. Fourth, PVL data was compared with fluoroscopic and echocardiographic data which are based on visual assessment and circumferential percentage, respectively. These estimates themselves are prone to significant error both due to interpreter variability and due to imaging modality limitations. Further assessment should be compared against either *in-vitro* flow models, *in-vivo* flow models in animals, or magnetic resonance imaging validated flow computations.

## Conclusion

In conclusion, we have developed a 3D printed and computational approach to predicting post procedure PVL in TAVR patients. Study approaches were complimentary and were capable of accurately predicting PVL. More importantly, we show that diseased native valve leaflet anatomies play a significant role in reducing PVL in patients undergoing TAVR placement. While more work needs to be done prior to using these techniques in a clinical setting, with further development this approach might be useful predicting development of post procedural PVL, ultimately improving patient selection, selecting the optimal prosthetic valve, and/or preparing for post procedural interventions.

## Data Availability

The raw data supporting the conclusion of this article will be made available by the authors, without undue reservation.

## Abbreviations

BASILICA, bioprosthetic or native aortic scallop intentional laceration to prevent iatrogenic coronary artery obstruction; CFD, computational fluid dynamics; DICOM, digital imaging and communications in medicine; LV, left ventricle; LVOT, left ventricle outflow tract; PVL, paravalvular leak; SAVR, surgical aortic valve replacement; STL, stereolithography; TAVR, transcatheter aortic valve replacement
